# Unveiling the Pathological Mechanisms of Death Induced by SARS-CoV-2 Viral Pneumonia

**DOI:** 10.3390/microorganisms12030459

**Published:** 2024-02-24

**Authors:** George-Călin Oprinca, Cosmin-Ioan Mohor, Alexandra Oprinca-Muja, Adrian Hașegan, Adrian-Nicolae Cristian, Sorin-Radu Fleacă, Ioana Boeraș, Roxana Cardoș, Diter Atasie, Manuela Mihalache, Cosmin Mihalache, Elena Teodora Tâlvan, Călin-Ilie Mohor

**Affiliations:** 1 Faculty of Medicine, Lucian Blaga University of Sibiu, 550169 Sibiu, Romania; georgecalin.oprinca@ulbsibiu.ro (G.-C.O.); lilioaraalexandra.muja@ulbsibiu.ro (A.O.-M.); adrian.hasegan@ulbsibiu.ro (A.H.); adrian.cristian@ulbsibiu.ro (A.-N.C.); radu.fleaca@ulbsibiu.ro (S.-R.F.); atasie.diter@ulbsibiu.ro (D.A.); manuela.mihalache@ulbsibiu.ro (M.M.); cosmin.mihalache@ulbsibiu.ro (C.M.); elena.talvan@ulbsibiu.ro (E.T.T.); calin.mohor@ulbsibiu.ro (C.-I.M.); 2 Faculty of Sciences, Lucian Blaga University of Sibiu, 550012 Sibiu, Romania; ioana.boeras@ulbsibiu.ro; 3 Department of Clinical Psychology, Babeș-Bolyai University of Cluj Napoca, 400347 Cluj-Napoca, Romania; roxana.cardos@ubbcluj.ro

**Keywords:** SARS-CoV-2, pneumonia, death, autopsy, histopathology, immunohistochemistry, molecular, RT-qPCR, pathology

## Abstract

In this comprehensive study of 15 deceased patients with confirmed SARS-CoV-2 infection, spanning a time frame of 1 to 68 days from confirmation to death, autopsies were meticulously conducted with stringent safety measures. Clinical, laboratory, histopathological, and molecular analyses were integrated, shedding light on diverse pulmonary lesions, including acute inflammatory changes, vascular abnormalities, and aberrant regenerative processes. Immunohistochemical analysis, utilizing various markers, successfully identified the SARS-CoV-2 nucleocapsid antigen within infected tissue cells and also revealed what type of inflammatory cells are involved in COVID-19 pathogenesis. Molecular investigations through rt-qPCR revealed the persistent presence and varying quantities of viral genes, even after 68 days. Moreover, the viral nucleocapsid was present even in patients who died after 50 days of infection onset. Employing statistical analyses such as Chi-square and phi coefficient tests, significant associations among microscopic lesions and their correlation with molecular and immunohistochemical findings were elucidated. We could draw a map of what kind of lesions were a direct consequence of viral invasion and what lesions where secondary to the acute immunological response. This integrative approach enhances our understanding of the intricate relationships between pathological features, providing valuable insights into the multifaceted landscape of COVID-19 pathogenesis.

## 1. Introduction

Ever since the outbreak of the COVID-19 pandemic, researchers have been trying to figure out all the pathological mechanisms behind this new viral infection that brought the whole world to a standstill. Even if the outbreak is over and the number of reported cases is constantly dropping, many people around the world have died or are suffering from irreversible damage caused by the SARS-CoV-2 virus and the chances of a new outbreak are extremely high. Also, like the original SARS-CoV virus or the MERS-CoV virus, SARS-CoV-2 jumped from animals to humans in a very aggressive manner and a repetitive leap with another coronavirus species remains a constant probability. Furthermore, as the viral morphology, pathogenesis, and acute phase clinical and pathological alterations are known in a relatively high percentage, this cannot be said about the fulminant cases with a high death rate or the subacute or “chronic” phase, termed long-COVID by clinicians, with patients suffering from irreversible organ damage and a whole batch of non-specific symptoms. Last but not least, it is essential to provide researchers, pathologists, and medical practitioners with multiple pathological patterns of disease depending on each type of case. Our study is based on 15 autopsies performed on patients who died at the County Clinical Emergency Hospital of Sibiu as a direct or indirect result of COVID-19, and the aim was to do so by drawing a pathological map of the SARS-CoV-2 infection, at a pulmonary level, by assessing lung tissues using histopathology, immunohistochemistry, and molecular analysis and comparing all individual results among themselves and also with the age and gender of the patients, comorbidities, and the time frame between the onset of symptoms and death, thus establishing the pathological patterns of SARS-CoV-2 infection. First, we have to review what is already known in the scientific community regarding the structure and pathogenesis of the novel coronavirus.

The SARS-CoV-2 virus, integrated into the Coronaviridae family, Betacoronavirus genus, Sarbecovirus subgenus, is an enveloped, single-stranded (ss) positive-sense RNA virus, related to the SARS-CoV and MERS-CoV betacoronaviruses that have also caused outbreaks in past years [1,2,3,4]. The structural components of the virion include the N-protein (nucleocapsid), which has the ability to recognize and attach to the viral genome. The C-terminal domain of the N protein can self-assemble and then mediate the formation of the N-protein tetramer, completing the formation of the helical structure of the viral nucleocapsid. In addition to providing a structural role, the N-protein underlies immunological evasion mechanisms mainly by antagonizing RNA interference (RNAi) and inhibiting interferon production [5]. The M-protein (membrane) consists of an N-terminal amino domain, a transmembrane domain (TMD), and a C-terminal carboxyl domain with the main purpose of determining the shape of the viral envelope [6]. The E-protein (envelope) composed of 75 amino acids contains an N-terminal transmembrane (TM) domain followed by a C-terminal domain and has a key role in viral assembly and immune evasion [7]. The S-protein (spike) contains two subunits (S1 and S2) located on the surface of the envelope, having a crucial role in viral cellular entry. The spike protein uses the ACE2 receptors (angiotensin conversion enzyme 2) to enter the host cell. After effective binding to the receptor, the spike protein undergoes conformational changes mediated by cell surface proteases such as TMPRSS2 and lysosomal proteases such as cathepsin—changes required for the fusion process [1,2,3,4,8]. In addition to these four structural proteins, the viral genome also encodes several nsps (non-structural proteins) via the ORF (open reading frame) genes with important roles in the pathogenesis of infection and immune evasion, for example, antagonizing interferon secretion, correction of the viral genome, protein adhesion, blocking of host RNA translation, promotion of cytokine expression, or cleavage of viral polyproteins [9,10].

After the first contact with a human host, the SARS-CoV-2 virus can generate two types of infection, both targeting the respiratory system. The first and most frequent is an upper respiratory tract infection, with common cold-like symptoms where the virus infects the ciliated cells in the nasopharynx, trachea, or bronchi or the sustentacular cells in the nasal mucosa [11]. In many cases, the immune system promptly stops the infection at this level. However, in a few cases, the virus is able to evade the defensive host responses and propagate deep in the pulmonary parenchyma and infect type II pneumocytes, lung endothelial cells, and alveolar macrophages [12]. The key elements that are able to stop the infection in the first phase are the early immunological responses mediated through interferon signaling and cytokine production by infected or bystander epithelial cells or local neutrophils and macrophages that induce an early adaptive immune response by T and B lymphocytes [11]. The first evasion tactics of the virus target these exact early stress signaling pathways of the host, mainly by blocking type I or type III interferon secretion by inhibiting IRF3 (interferon-regulatory factor) activation and translocation, blocking the production and function of PRRs (pattern recognition receptors), the inhibition of interferon synthesis, and transmembrane transport with the help of nsps [13]. The literature also describes some cases in which the primary site of infection is the lower respiratory tract by direct invasion of the type II pneumocytes [11].

## 2. Materials and Methods

We evaluated 15 patients admitted to the hospital after they had been confirmed with SARS-CoV-2 infection and who eventually died as a direct result of COVID-19 disease. The time frame between the confirmation of infection and death was between 1 and 68 days. We carefully assessed all clinical and laboratory evaluations of the patients while hospitalized. All autopsies were performed in the COVID-19 restricted area of the county morgue using complete protective equipment and following all national protocols in effect. After a careful macroscopic examination of the body and internal organs, we conceived a detailed description of the anomalies found and collected tissue samples from multiple pulmonary sites for histopathological examination. We also sampled tissue for the detection of SARS-CoV-2 viral RNA utilizing rt-qPCR (reverse transcriptase polymerase chain reaction) molecular investigations and the detection of viral nucleocapsid using SARS-CoV-2 monoclonal antibody with the help of immunohistochemical analysis.

Tissue samples collected for histopathology were fixed in a 10% formaldehyde solution. Following fixation and dehydration, the samples were embedded in blocks of paraffin. Using a microtome, tissue incorporated into the paraffin wax was cut and mounted on microscopic slides. After the dewaxing, the slides were colored with the classic hematoxylin–eosin dye, like in previous studies we conducted [14].

The immunohistochemical preparation followed the same protocol as in previous research conducted in our laboratory. The method for antibody incubation was automated, utilizing the Epredia Autostainer 360 (Epredia, Kalamazoo, MI, USA). Tissue specimens embedded in paraffin wax were manually cut using a microtome to a thickness of 3–5 microns. Subsequently, these sections were mounted on positively charged microscopic slides to ensure stable adhesion. The slides containing the cut tissue specimens were incubated for 2 h at 58 °C. Before epitope demasking, the slides underwent deparaffinization with xylene and rehydration using ethanol in decreasing concentrations. Epitope recovery was facilitated using the ImmunoDNA Retriever kit (BioSB, Santa Barbara, CA, USA) with a citrate buffer solution at a pH level of 6. The primary antibodies used originated from the IgG mouse monoclonal isotype. This set included the SARS-CoV-2 nucleocapsid antibody for detecting the viral nucleocapsid within the cytoplasm of infected cells. Additional immunostains comprised the CD3 and CD5 antibodies for detecting T lymphocytes and natural killer cells, CD20 for B lymphocytes, and CD68 for cells of the mononuclear phagocyte lineage. These immunomarkers were employed in our study to comprehensively assess the immunological reaction within the lung infected tissue. We also utilized cytokeratin immunomarkers such as CK MNF 116 and CK7 to evaluate type II pneumocytes and diagnose type II pneumocyte hyperplasia. The tyrosine transcription factor I (TTF1) played a crucial role in evaluating and quantifying the type II pneumocytes. These three markers were particularly significant in detecting the origin of multinucleated giant cell-like aggregates consistently found within infected lung tissue specimens. CKAE1/AE3 was used for quantifying the hyaline membranes, which we observed to be positive for this immunomarker. All selected antibodies were ready to use, eliminating the need for a dilution protocol. For the chromogenic immunohistochemical reaction, we employed an anti-mouse HRP IgG secondary antibody with DAB chromogen. Hematoxylin was used for counterstaining [15].

For the molecular analysis, RNA extraction from samples preserved in RNA lock reagent at −20 °C was carried out using the QIAamp viral RNA extraction kit (Qiagen, Hilden, Germany) following the manufacturer’s guidelines. RNA elution was performed in a final volume of 60 μL elution buffer. To validate the RNA extraction process and ensure the absence of inhibitors in the RT-qPCR reactions, in vitro-generated MS2 RNA was introduced into the sample lysis buffer as a control. MS2, also known as Emesvirus Zinderi bacteriophage, is an icosahedral, positive-sense single-stranded RNA virus frequently used as a control for molecular analysis, indicating proper RNA extraction and amplification. The presence and quantity of the N (nucleocapsid), S (spike), and ORF1ab SARS-CoV-2 viral genes, along with the MS2 control, were determined through reverse-transcription quantitative PCR using the TaqPath COVID-19 CE-IVD RT-PCR kit (Thermo-Fisher Scientific, Waltham, MA, USA). The reverse transcription and quantitative PCR were conducted with the following reaction mix: 6.25 μL TaqPath™ 1-Step Multiplex Master Mix (No ROX™) (4×), 1.25 μL COVID-19 Real-Time PCR Assay Multiplex, 7.5 μL nuclease-free water, and 10 μL RNA. After preparation, the probes were incubated for 2 min at 25 °C with the uracil-N-glycosylase enzyme, followed by incubation for 10 min at 53 °C for reverse transcription and 2 min at 95 °C for activation. Finally, the probes underwent 40 cycles of 3 s at 95 °C for denaturation and 30 s at 60 °C for annealing and extension, following the manufacturer’s recommendations (Thermo-Fisher Scientific). The primers and probes in the kit, designed and validated by Thermo-Fisher Scientific, were approved for the detection of genes from the SARS-CoV-2 viral genome. Samples were considered positive for N, S, or ORF1ab gene targets if the threshold cycle (Ct) was below 40 cycles. The limit of detection for the three primers and probes sets in the TaqPath COVID-19 CE-IVD RT-PCR kit was determined by the producer to be 10 copies of viral RNA per reaction, and similar results were obtained in our laboratory.

All microscopic slides were digitalized with the help of a Pannoramic Desk II DW digital slide scanner (Budapest, Hungary) and examined using the 3DHistech Slide Viewer application Version 2.7. Photographs were taken in UHD (ultra-high definition) using the same application.

We performed Chi-square and phi coefficient statistical analysis for dichotomous variables to confirm the most significant connections between various microscopic lesions and between these microscopic abnormalities and the molecular and immunohistochemical results. The phi coefficient is a statistical measure used to quantify the strength and direction of association between two categorical variables. The study aims to uncover potential relationships between the variables under investigation by calculating the phi coefficient. The interpretation of the phi coefficient involves discerning whether the variables are positively, negatively, or not significantly correlated. A value close to +1 signifies a strong positive correlation, while a value near −1 indicates a strong negative correlation. A phi coefficient close to 0 suggests a lack of correlation between the variables. Through this analysis, the research seeks to enhance our understanding of the interconnectedness of the categorical variables and contribute valuable insights to the broader scope of the study.

## 3. Results

Out of our 15 patients who died following a confirmed SARS-CoV-2 infection, we determined with the help of histopathology, immunohistochemistry, and rt-qPCR analysis that in the case of 9 patients, the direct cause of death was an acute respiratory distress syndrome triggered by viral pneumonia, with 4 of them also having chronic pulmonary lesions translated by marked pulmonary fibrosis in association with viral persistence. In the case of three patients, tissue analysis revealed abnormalities related to COVID-19 viral pneumonia but with alterations suggesting a bacterial superposition in a relatively short time frame before death (Table 1). One 68-year-old female patient died after a spontaneous retroperitoneal hemorrhage following anticoagulation therapy but with microscopic and immunohistochemical changes related to viral pneumonia and pulmonary fibrosis. Our youngest patient, a 31-year-old male, died after 68 days from the confirmed nasopharyngeal swab test and after 39 days of admission, with the cause of death being severe sepsis secondary to multiple bacterial infection of the respiratory tract and supra-infected decubitus ulcers with multidrug-resistant bacteria. The last case, a 71-year-old female died after a massive pleural effusion associated with a stage IV pulmonary neoplasm but with histopathology and immunohistochemistry revealing changes related to SARS-CoV-2 pulmonary involvement (Table 1).

To fully understand the pathological changes in the novel viral infection, it is of great importance to determine the time pattern of these lesions and to do so we had to evaluate patients with different time intervals between the onset of symptoms, respectively, a first positive nasopharyngeal swab rt-qPCR test, and death. From our group of 15 patients, 5 patients had been confirmed with the infection 1 to 3 days before death, 2 patients died 4–7 days after the confirmation of infection, 4 patients presented symptoms and a confirmation test between 15 and 21 days before death, and 4 patients died 21 days after PCR confirmation, the longest time interval being 68 days (Table 1).

**Figure 1:**
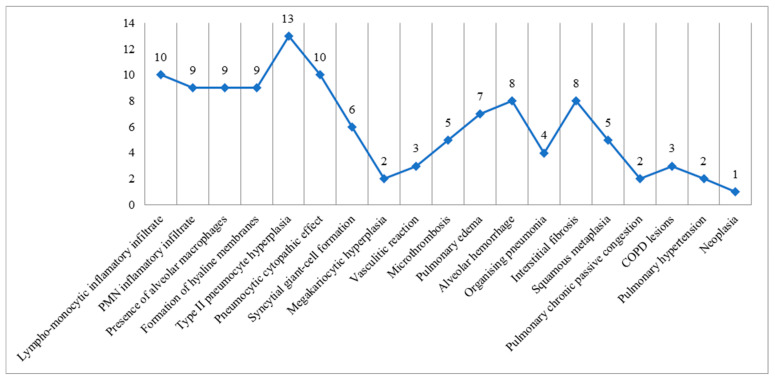
Graphic representation of histopathological lesions observed in the study sample lung specimens.

After all autopsies were performed, every sample collected was subjected to histopathology using the classic hematoxylin–eosin stain, immunohistochemical analysis using SARS-CoV-2 nucleocapsid antibody, and rt-qPCR molecular analysis for the detection of the S, N, and ORF1ab genes. We found the most significant changes in the pulmonary tract and, as such, the histopathology of lung tissue sampled from every case revealed the following (Figure 1):

- Acute inflammatory lesions:
  ○ Lympho-monocytic inflammatory infiltrate: 10 cases;
  ○ Polymorphonuclear (mostly neutrophils) inflammatory infiltrate: 9 cases;
  ○ Mixed inflammatory infiltrate: 4 cases;
  ○ Presence of macrophages in the alveolar space: 9 cases.
- Acute alveolar lesions
  ○ Hyaline membrane formation: 9 cases;
  ○ Type II pneumocyte hyperplasia: 13 cases;
  ○ Pneumocytes with cytopathic effect: 10 cases;
  ○ Multinucleated giant cell-like pneumocyte aggregates: 6 cases;
  ○ Megakaryocyte hyperplasia: 2 cases.
- Vascular lesions
  ○ Vasculitic reaction: 3 cases;
  ○ Microthrombosis: 5 cases.
- Hemodynamic injuries
  ○ Pulmonary edema: 7 cases;
  ○ Diffuse alveolar hemorrhage: 8 cases.
- Interstitial and aberrant regenerative alterations
  ○ Organizing pneumonia: 4 cases;
  ○ Interstitial fibrosis: 8 cases;
  ○ Squamous metaplasia: 5 cases.
- Associated chronic injury
  ○ Chronic passive congestion of the lung: 2 cases;
  ○ COPD (Chronic obstructive pulmonary disease) changes: 3 cases;
  ○ Vascular changes secondary to pulmonary hypertension: 2 cases;
  ○ Neoplasm: 1 case.

The majority of cases of inflammatory infiltrate were attributed to the presence of lymphomonocytic infiltrate, observed in 10 cases. This infiltrate exhibited either a focal disposition, with areas of lymphocytes or monocytes alternating with non-inflamed areas, or a diffuse disposition, involving the entire analyzed parenchyma to varying extents. Both focal and diffuse forms were near equally represented in the study group; the first was found in five cases and the second observed in six cases. Afterwards, the inflammatory infiltrate was subdivided into either a rich infiltrate or a poor inflammatory process. Infiltrate classification as rich or reduced was based on inflammatory cell digital quantification, where a rich infiltrate constituted over 50% of the histological structure. Five cases showed a rich lymphomonocytic infiltrate with a focal disposition, while four cases exhibited a rich infiltrate with a diffuse disposition. Cases with a mildly augmented lymphomonocytic infiltrate were fewer, with only two cases in focal disposition. The focal infiltrate was more perivascular, peribronchial, interalveolar, interlobular, or subpleural, while the diffuse infiltrate extended widely through the lung parenchyma. Most of the lymphocytes expressed CD3 positivity. Among the 15 patients, 9 of them exhibited a more predominantly neutrophilic polymorphonuclear infiltrate. Quantitatively, there was a relatively equal distribution among subcategories, with two cases having a rich focal polymorphonuclear infiltrate, four cases having a reduced focal infiltrate, and three cases showing a rich diffuse polymorphonuclear infiltrate. Unlike the lymphocytic infiltrate, the polymorphonuclear infiltrate was predominantly alveolar but also involved interalveolar septa, interlobular septa, and perivascular areas. Another category of inflammatory lesions involved acute phase macrophages in alveolar spaces, observed in nine patients. This was confirmed by CD68 immunostaining.

**Figure 2:**
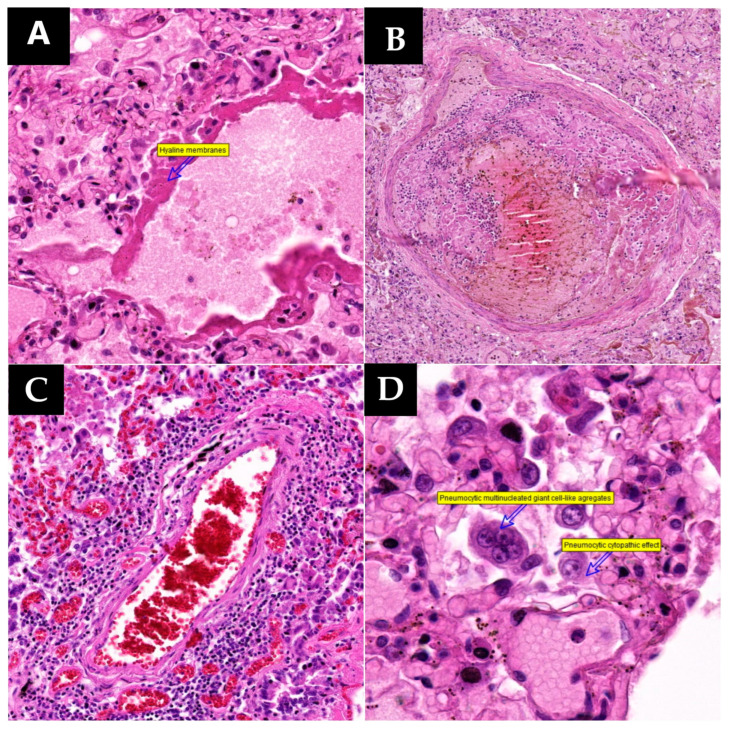

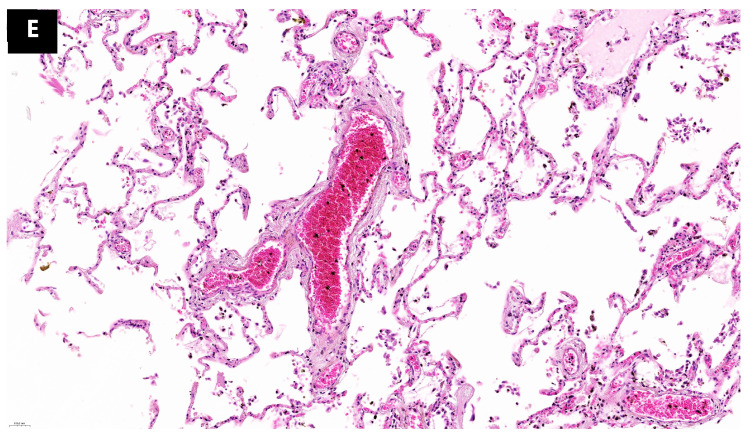
Lung samples collected (HE staining): (**A**) (220.2×)—Hyaline membrane formation; (**B**) (60.4×)—Microthrombosis of a pulmonary artery; (**C**) (140.6×)—Lymphocytic vasculitis; (**D**) (480.4×)—Pneumocyte hyperplasia with cytopathic effect and multinucleated giant-cell formation. (**E**) (230.1×)—Normal control specimen presenting clear alveolar spaces and a normal blood vessel.

In specimens collected from 13 patients, histopathological examination revealed changes secondary to alveolar lesions, with the most common being type II pneumocyte hyperplasia. Among patients with suggestive histopathological changes in type II pneumocyte hyperplasia, 10 exhibited variable proportions of hyperplastic pneumocytes with cytopathic effects (Figure 2). Cytopathic effects result from the invasion of a cell, typically by a cytopathic virus, leading to morphological, physiological, biological, and genetic alterations. Virally infected cells undergo shape and size changes, dysfunction of the cytoskeleton, and secondary nuclear changes and often display cytoplasmic or nuclear inclusions formed by newly formed virions or their structural proteins [16].

In six collected specimens showing pneumocyte hyperplasia with viral cytopathic effects, altered cells occasionally fused to form syncytia, referred to as pneumocytic aggregates with giant cell-like formation or syncytial giant cell-like aggregates (Figure 2). These can be easily mistaken for multinucleated giant cells of histiocytic origin. This was confirmed by CF7 and TTF1 positive immunostaining and CD68 negative immunostaining. Syncytium formation involves cell-to-cell fusion and is commonly observed in infectious processes. The ability of viruses to mediate membrane fusion for host cell invasion is employed for transmembrane transfer between infected donor cells and neighboring uninfected cells, facilitating local–regional expansion and potentially contributing to syncytium formation. The formation of syncytia is primarily mediated by specific interactions between viral fusion proteins and surface molecules or receptors expressed on adjacent uninfected cells [17]. Giant-like cells in autopsy specimens exhibit wide aggregates with eosinophilic cytoplasm, abundant large nuclei, round-oval, irregular, homogeneous chromatin, predominantly peripheral nuclear disposition, visible nucleoli, disordered nuclear arrangement without a specific pattern, and occasional discernible boundaries between fused cells.

Another significant histopathological change within the spectrum of diffuse alveolar lesions is the formation of hyaline membranes, observed in nine cases. These membranes consist of dense, intensely eosinophilic, acellular bands on the inner surface of alveolar walls, composed of pneumocytic debris and fibrin (Figure 2). 

Addressing acute changes, vascular lesions, including microthromboses and vasculitic reaction, were observed in the current cases. Microthromboses, extensively discussed in the literature, underscore the need for anticoagulant treatment in severe COVID-19 pneumonia. Hence, this study emphasized the inclusion of tissue specimens from diverse pulmonary areas for effective vascular evaluation, covering broad lung fields. Vascular thrombi, of either small or large caliber, were observed in five cases (Figure 2). In contrast, vasculitic reaction was noted in only three patients. Histologically, the vasculitic reaction did not manifest as classic leukocytoclastic vasculitis with secondary fibrinoid necrosis. Instead, it exhibited a lymphocytic nature, with lymphocytic groups penetrating the vascular wall from the adventitia toward the vascular endothelium (Figure 2). Among these patients, only one presented both types of vascular lesions.

Histopathological examination revealed a series of hemodynamic changes, primarily characterized by pulmonary edema and diffuse alveolar hemorrhage. Accordingly, seven cases exhibited pulmonary edema, while eight cases showed diffuse alveolar hemorrhage. Among these, five patients presented both pulmonary edema and diffuse alveolar hemorrhage, and ten patients exhibited at least one form of these two conditions.

**Figure 3:**
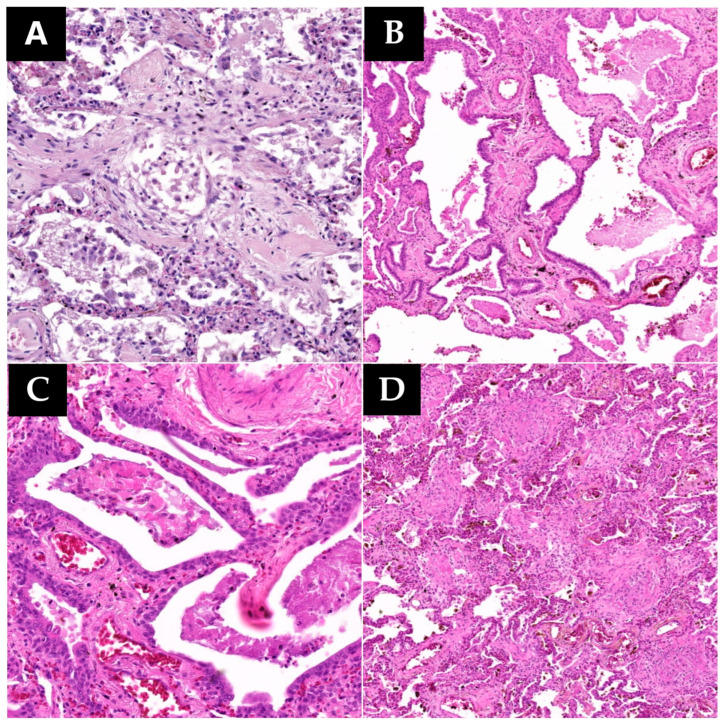

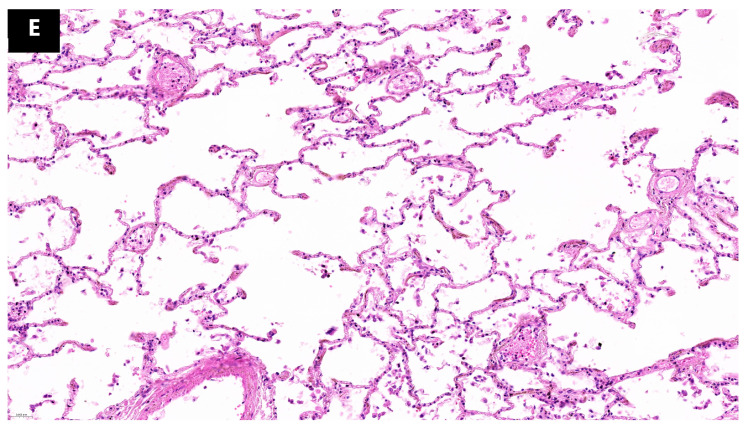
Lung samples collected (HE staining): (**A**) (120.3×)—Organizing pneumonia; (**B**) (50.7×)—Squamous metaplasia with extensive fibrosis; (**C**) (140.5×)—Squamous metaplasia of the alveolar wall; (**D**) (50.7×)—Intense interstitial fibrosis. (**E**) (250.2×)—Normal control lung specimen highlighting clear alveolar spaces and alveolar walls of normal thickness.

During histopathological examination, numerous microscopic lesions in the pulmonary tissue corresponding to alterations in remodeling and tissue regeneration processes were observed, likely indicative of a prolonged evolution of SARS-CoV-2 infection. These lesions, termed aberrant regenerative changes, encompassed organizing pneumonia, interstitial fibrosis, and alveolar squamous metaplasia (Figure 3). Aberrant regenerative lesions succeeded the acute lesions of the exudative phase, including hyaline membranes, type II pneumocyte hyperplasia, alveolar hemorrhage, and inflammatory infiltrate, entering the spectrum of the proliferative organization phase within acute alveolar lesions [18]. Thus, the study identified eight cases exhibiting at least one form of aberrant regenerative change. Among these, four cases presented organizing pneumonia, eight cases showed interstitial fibrosis, and five cases exhibited squamous metaplasia. Four cases presented all three forms described, one case presented two of the three forms, and three cases presented only one aberrant regenerative lesion. Correlating these findings with the time interval between the onset of symptoms and death, we observed that one out of three described lesions tend to emerge after a few days following infection (between 2 and 21 days) and after 23 days all three lesions described can be detected.

Molecular analysis of pulmonary tissues collected from every autopsy and stored in RNA lock reagent for ARN extraction revealed a high positivity rate for all three SARS-CoV-2 genes, namely 14 out of 15 cases. One case presented positivity for two out of three genes, namely the N and ORF1ab genes (Table 2).

**Figure 4:**
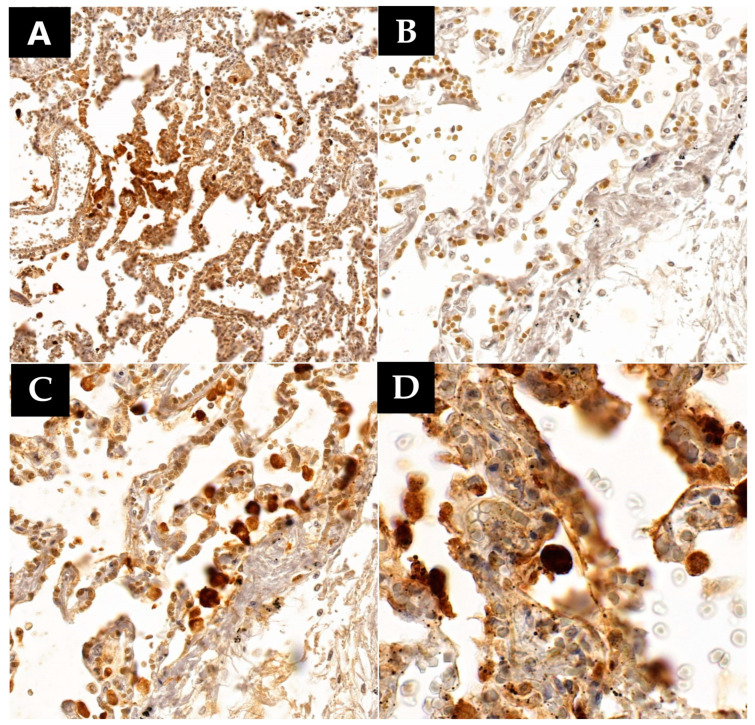
Lung samples collected (SARS-CoV-2 nucleocapsid immunostaining): (**A**) (110.0×)—Intense diffuse positivity in lung parenchyma; (**B**) (230.4×)—Negative control; (**C**) (230.4×)—Intense positivity in pneumocytes (from the same area as (**B**)); (**D**) (500.4×)—Intense positivity in type II pneumocytes.

Immunohistochemistry also revealed a high positivity count in lung tissue, translated by the presence of the nucleocapsid protein in 12 out of 15 cases (Table 3), mostly found at the level of the type II pneumocytes and the multinucleated giant-cell like aggregates (all 12 cases), in the alveolar macrophages (9 cases) or fibroblasts (6 cases), and in the hyaline membranes in the cases where this membrane formation was observed (5 cases) (Figure 4). Regarding the pneumocytes, seven cases exhibited extensive positivity, surpassing one pneumocyte per microscopic field at 400× magnification, while in five cases, the positivity was less extensive, below one infected cell per microscopic field at 400× magnification (Figure 4). Since the detection of the nucleocapsid by the specific antibody induces a cell-staining reaction, and the staining intensity is directly proportional to the number of proteins in the cell, a more intense staining suggests a higher nucleocapsid presence. Each evaluated specimen was quantified based on the staining intensity of the infected cells. Of the seven cases with extensive positivity (>1 cell/400× field) for the anti-nucleocapsid antibody at the pneumocytic level, all cases exhibited intense positivity in each infected cell. All these patients succumbed directly to acute respiratory distress syndrome (ARDS) secondary to severe viral pneumonia. Among them, four had a confirmed infection onset between 1 and 7 days before death, one patient passed away 15 days after infection confirmation, and the last two patients died after 21 or 28 days post-SARS-CoV-2 infection confirmation. In the group positive for less than one cell/400× microscopic field (five cases), a total of three cases exhibited intense positivity to the same antibody. The first of these three patients died one day after infection confirmation from ARDS secondary to viral pneumonia. The second patient succumbed 23 days after infection onset, also from severe pulmonary lesions associated with viral pneumonia. The last patient from this group died after 15 days of infection confirmation, with the cause of death being bacterial bronchopneumonia. The last two cases showed only weak focal positivity to the specific antibody. One of them died after 7 days of infection onset due to a massive pleural effusion. This patient had very few lesions consistent with viral pneumonia, such as a high number of macrophages within the alveolar spaces and type II pneumocyte hyperplasia, but also focal pulmonary fibrosis. Instead, this patient presented a pulmonary neoplasm triggering a massive pleural effusion in association with mild viral pneumonia. The second patient died 68 days after infection onset from a massive pulmonary edema, most likely secondary to acute cardiac insufficiency. The patient was hospitalized for almost the entire time, surviving severe viral pneumonia with multiple bacterial superinfections. The last three patients from our study group were all negative for the SARS-CoV-2 nucleocapsid, and death was triggered either by bacterial superinfection or spontaneous retroperitoneal hemorrhage secondary to anticoagulant therapy. The time span between infection confirmation and death in these last three patients ranged from 1 to 25 days. Upon comprehensive analysis of these results, a pattern emerges. The nucleocapsid is extensively present in large quantities in patients who develop severe diffuse alveolar damage in the first three weeks of infection. After three weeks, the nucleocapsid is still present but more focally and in lesser quantities. However, it can still be detected in small quantities even after 68 days after infection onset, even in patients who no longer exhibit lesions consistent with acute viral pneumonia.

The other antibodies used in this study, including CD3, CD5, CD20, CD68, CK7, TTF1, CK AE1/AE3, and CK MNF 116, were employed solely to elucidate the exact origin of cells involved in the pathological mechanism of SARS-CoV-2 viral pneumonia. Through this approach, we can confidently affirm that the cells sustaining viral cytopathic effects in the examined lung tissues are of pneumocytic origin, specifically type II pneumocytes positive for CK7, TTF1, and CK MNF 116. We have conclusively ruled out the possibility that the multinucleated giant cells found were of macrophagic origin, as these cells tested negative for CD68 but positive for CK7, TTF1, and CK MNF 116, indicating a pneumocytic origin. The majority of hyaline membranes developed within diffuse alveolar damage exhibited intense positivity for pancitokeratin, confirming that these membranes are formed by abundant cellular debris secondary to alveolar wall epithelial destruction. Almost all lymphocytic lung infiltrate was positive for CD3 and CD5, with only scattered lymphocytes positive for CD20, indicating that T lymphocytes are the main immunological cells of lymphocytic origin involved in the pathogenesis of SARS-CoV-2 infection within the lung tissue (Table 4).

After performing the statistical analysis, we discovered several significant correlations between our main findings. Patients with diffuse alveolar damage with hyaline membrane formation were at higher risk of developing a diffuse alveolar hemorrhage immediately before death, with a chi-square of 5.402 and a phi coefficient of 0.600 (*p* value of 0.02). Regarding the correlation between the immunohistochemical results and the most important microscopic alterations, we found a significant link between the formation of pneumocyte syncytial giant cells and the diffuse and intense presence of the viral nucleocapsid at the level of the pneumocytes with a chi-square of 5.402, a phi coefficient of 0.600, and a *p* value of 0.002. This correlation indicates that the pathophysiological mechanism behind the formation of these multinucleated syncytial giant cells is triggered by the direct invasion of the SARS-CoV-2 virus of the type II pneumocytes.

## 4. Discussion

In the current study cohort (N = 12), viral pneumonia was the main culprit in triggering the mechanism of death (N = 9), followed by bacterial superinfections (N = 4). A prospective study on a sample of 735 deaths associated with SARS-CoV-2 infection, published at the end of 2021, determined that the primary cause of death among the studied patients was viral pneumonia, accounting for 73.6%, followed by pulmonary thromboembolism at 9.4%, and cardiac injuries in 5.9% of cases [19]. Another study concluded that viral pneumonia was established as the primary cause of death in 76.2% of cases [20]. Massive hemorrhage was present in only one patient, represented by spontaneous retroperitoneal hemorrhage into the iliopsoas muscle during anticoagulant treatment. In this case, all three viral genes were exclusively detected at the pulmonary level, while the viral nucleocapsid was absent. The final patient succumbed to a massive pleural effusion amid stage IV lung adenocarcinoma. Molecular examinations unveiled the presence of only two out of the three studied viral genes in each autopsy specimen, and immunohistochemical analysis confirmed the occurrence of viral nucleocapsid in a diminished proportion within the lungs. Another frequent complication of SARS-CoV-2 infection that can cause severe multiorgan damage and death is Clostridium Difficile enterocolitis, but recent treatments can decrease the mortality rate, mainly through fecal microbiota transplantation [21].

Regarding the number of days between the onset of the disease and death, the distribution was variable, with the majority falling within the 1–3 days interval, encompassing five cases. Next, there were four cases within the 15–21 days range and over 21 days, respectively, while only two cases were observed in the 4–7 days interval. In one male patient, we noticed a maximum time span—from symptomatic onset to death—of 68 days. Death occured after a massive pulmonary edema. In conclusion, the average time interval between infection onset and death was 15.3 days. These findings suggest that the risk of death plateaus in the initial days after onset, except for the cases with rapid progression (1–3 days), followed by an increase after 15 days due to a slow and unfavorable evolution associated with various complications. Similar results have been observed in the specialized literature, albeit with a narrower range compared to the current study. Specifically, Verity R. et al. established an average of 17.8 days between symptomatic onset and death [22]. Baud D. et al. outlined a range of 2 to 8 weeks as having the highest incidence of death in confirmed SARS-CoV-2-infected patients [23]. Madabhavi I. et al. investigated deaths with an infectious onset-to-death interval ranging from 6 to 41 days, with an average of 14 days [24]. Qiurong R. et al. noted two peaks in the incidence of deaths, one at 14 days and another at 22 days from the onset of infection [25].

Within the lung, the entire spectrum of lesions identified through microscopic examination was classified into three major categories: acute lesions (the most numerous), aberrant regenerative changes, and associated chronic lesions. Within acute lesions, we observed various types of microscopic changes, encompassing the spectrum of inflammatory or alveolar lesions, as well as vascular or hemodynamic lesions. Inflammatory lesions involved the presence of inflammatory infiltrate in the pulmonary parenchyma, either interstitial or alveolar. This was categorized as either lymphomonocytic, polymorphonuclear, or mixed, with the macrophagic infiltrate always overlapping with the first.

Within alveolar lesions, we noticed a degree of type II pneumocyte hyperplasia in 13 out of 15 cases, and the immunohistochemical analysis with anti-TTF1 and CK7 antibodies confirmed the cellular origin. The cytopathic effects of these pneumocytes were noted in 10 cases, followed by the presence of hyaline membranes observed in 9 cases. Multinucleated giant cells were present in 6 cases, and immunohistochemical analysis demonstrated their pneumocytic origin, as they were positive for TTF1 and CK7 antibodies and negative for CD68. Also, these cells presented intense positivity for the anti-nucleocapsid antibody, confirming the direct invasion of the virus within these cells. These described inflammatory and alveolar lesions constitute the histopathological pattern of diffuse alveolar damage in the exudative and proliferative phases, clinically manifested as acute respiratory distress syndrome (ARDS). This syndrome is commonly diagnosed in patients with severe COVID-19 pneumonia [26,27,28]. Moreover, the presence of these destructive lesions of the alveolar wall, with detachment of the surface epithelial lining replaced by necrotic cellular debris forming hyaline membranes and reactive type II pneumocyte hyperplasia, confirms the theory that the gas exchange deficit triggered by the loss of the alveolar-capillary membrane can be considered a significant pathophysiological mechanism through which the SARS-CoV-2 virus induces acute respiratory distress syndrome. These findings are supported by other specialized studies as well [29,30,31,32,33,34]. Syncytia are large multinucleated cells formed by the fusion of two or more cells, where the plasma membrane of different cells merges into a single double lipid layer along with their intracytoplasmic content [35,36]. Contemporary studies have demonstrated the presence of viral S protein on the surface of infected pneumocytes. This protein interacts with ACE2 receptors on the surface of neighboring cells, leading to fusion through the TMPRSS2 protease and the formation of multinucleated giant cells [37,38]. 

Vascular lesions were characterized by the presence of microthrombi in the lumen of small or medium-sized vessels in five cases and by a lymphocytic inflammatory reaction in the walls of small intrapulmonary vessels in three cases. The latter could not be classified as classic leukocytoclastic vasculitis due to the absence of a rich inflammatory infiltrate in the vascular wall and the absence of fibrinoid necrosis. However, the denudation of the vascular endothelium and the presence of lymphocytes around blood vessels penetrating the vascular wall suggest an immune reaction directed against vascular structures. Several studies in the specialized literature confirm the presence of these lesions, including Satturwar S. et al., who describe microthrombosis in 59% of the cases studied and vascular lesions such as lymphocytic endothelitis or capillaritis, also mediated by lymphocytes and without the presence of fibrinoid necrosis, in 21% of cases [39]. One theory in the literature suggests that the SARS-CoV-2 virus accumulates in pulmonary vessels, causing exudative vasculitis accompanied by the appearance of noncanonical monocytes expressing thrombospondin-1 and the formation of microthrombi containing myosin light chain 9 (Myl9) in the lungs of patients with severe COVID-19 [40].

Pulmonary edema was present in seven patients, and diffuse alveolar hemorrhage was observed in eight patients. The direct viral mechanism involved in triggering pulmonary edema is mediated by the M membrane protein of the SARS-CoV-2 virus. When alveolar epithelial cells are invaded by the microorganism, the M protein triggers mitochondrial apoptosis through BOK (Bcl-2 related ovarian killer), stabilizing it by inhibiting ubiquitination and promoting mitochondrial BOK translocation [41]. In the case of alveolar hemorrhage, as a consequence of severe COVID-19 pneumonia, injuries to the alveolar wall and a massive increase in capillary permeability trigger this type of lesion. Additionally, microangiopathic lesions such as lymphocytic capillaritis can lead to diffuse hemorrhages in the pulmonary parenchyma [42].

Among the aberrant regenerative lesions, the most frequent were extensive interstitial fibrosis (eight cases), followed by squamous metaplasia (five cases), and lastly, organizing pneumonia (four cases). The exact immunologic mechanism triggering pulmonary fibrotic processes in some patients is not fully understood, However, certain studies suggest that the major role in these processes is played by the exaggerated activation of monocyte-derived macrophages (CD163/LGMN-Mϕ) rather than tissue macrophages. The former interact extensively with fibroblasts and myofibroblasts, leading to the overstimulation of genes encoding extracellular matrix (ECM) [43]. In line with the current study analysis, we noticed an association between interstitial fibrosis and the massive presence of alveolar macrophages. Parimon T. et al. also suggest that the fibrogenic mechanism is largely induced by the presence of long-lived circulating monocytes in the pulmonary parenchyma stimulated by numerous cytokines, frequently observed in patients diagnosed with cytokine storm [44]. In the case of squamous metaplasia, we identify several signaling pathways activated in type II pneumocytes by secreted inflammatory factors, inducing the transformation of pneumocytic epithelium into squamous epithelium. These signaling pathways are much more expressed in SARS-CoV-2 infection when compared to other viral infections, such as influenza, demonstrating the increased specificity of this type of lesion in the context of COVID-19 pneumonia [45].

We detected the SARS-CoV-2 viral genome at the pulmonary level in 14 out of 15 cases. We could not demonstrate that the cause of death for the last case was viral pneumonia; therefore, excluding it, we can conclude that in patients where death was triggered by viral pneumonia, there was a 100% detection rate for the three studied viral genes. Detection could be achieved even after 68 days from the onset of infection. Similar results have been highlighted in the specialized literature. A study from Argentina observed increased positivity and persistence of the viral genome in the study cohort for up to 22 days from onset [46]. A cohort study demonstrates the presence of the viral genome in the lungs even after 67 days [26]. Another study confirms the presence of the viral genome even after 6 weeks from onset [47]. An interesting publication by Musso N. et al. managed to demonstrate the persistence of viral genetic material in exhumed corpses for up to 78 days after death [48].

Following immunohistochemical analysis using the anti-SARS-CoV-2 nucleocapsid antibody, detection was observed in 12 out of 15 cases. The most intense positivity was noted in pneumocytes, with detection being possible in type II pneumocytes, most of which exhibited viral cytopathic effects or the formation of syncytial giant cells in all 12 cases. Nucleocapsid was simultaneously present in macrophages in nine patients, in fibroblasts in six patients, and in hyaline membranes where they were present in five patients. Viral nucleocapsid was present in all cases where pneumocytes with viral cytopathic effects were observed and in all cases presenting pneumocytic syncytial giant cells. Although the detection of the nucleocapsid through immunohistochemistry was weak and focal, it could still be achieved even after 68 days from the onset of infection. Similar results regarding nucleocapsid detection in the lung and its persistence over an extended period have been reported in the specialized literature [49,50,51].

## 5. Conclusions

In the initial days of infection confirmation, the elevated detection of viral proteins and genome is observed in pneumocytes, macrophages, and fibroblasts, with heightened replicative activity. This triggers an oversaturated inflammatory response mediated by lymphomonocytes and polymorphonuclear cells, accompanied by numerous alveolar macrophages. The inflammatory response and constant viral replication initiate diffuse alveolar lesions in the exudative phase, characterized by massive pneumocytic destruction forming hyaline membranes and the marked hyperplasia of type II pneumocytes. Most of these changes result from viral invasion, inducing cytopathic effects or forming pneumocytic syncytial giant cells via the viral S protein. The exaggerated inflammatory phenomenon and ongoing viral replication lead to microvascular lesions. The invasion of macrophages expressing procoagulant factors activates the coagulation cascade, forming vascular microthrombi. The extension of alveolar and microvascular lesions reduces the gas exchange surface, triggering ARDS. Subsequently, due to increased vascular permeability or worsening microvascular lesions, diffuse alveolar hemorrhages or massive pulmonary edema may occur, potentially leading to death. A prothrombotic state may even induce a massive pulmonary embolism as a fatal mechanism. Surviving patients enter a stable immune response phase with a gradual reduction in viral replication, resulting in a slow evolution of the infection. Transitioning from the exudative to the proliferative phase, viral replication peaks in this proliferative stage and then gradually decreases in intensity. In this phase, inflammatory cell numbers decrease, but macrophages persist, activating fibroblasts and myofibroblasts that produce the extracellular matrix, initiating the extensive fibrotic phase of diffuse alveolar lesions. These lesions become increasingly prominent, with the viral nucleocapsid and genome still present in the pulmonary parenchyma, though viral replication is reduced. However, chronic inflammation persists, inducing ongoing lung injuries. Massive destruction of the pneumocytic lining during the acute phase leads to extensive squamous metaplasia of the alveolar epithelium, marked fibrosis, and the potential persistence of hyaline membranes. Patients enter a phase of progressive, chronic respiratory failure that may decompensate other organs and lead to death. Also, in this phase, patients could suffer severe complications like bacterial superinfection, sepsis, or massive hemorrhage.

## Figures and Tables

**Table 1:** General data of the study sample.

Case Number	Gender	Age	Number of Days from Infection Confirmation and Death	Cause of Death
1	M	68	1	Viral pneumonia
2	F	69	1	Viral pneumonia
3	M	85	2	Viral pneumonia
4	M	64	15	Bacterial superinfection
5	M	77	23	Viral pneumonia
6	F	82	15	Viral pneumonia
7	M	61	28	Viral pneumonia
8	M	31	68	Pulmonary edema
9	F	68	15	Spontane retroperitoneal hemorrhage
10	F	76	1	Bacterial superinfection
11	F	71	7	Marked pleural effusion
12	M	54	7	Viral pneumonia
13	F	58	25	Bacterial superinfection
14	M	62	21	Viral pneumonia
15	M	78	1	Viral pneumonia

**Table 2:** RT-qPCR results in the study group.

Case Number	Cycle Threshold (CT) Value			
	MS2 (Control)	N Gene	ORF1ab	S Gene
Case No. 1	32.651	28.140	27.811	28.061
Case No. 2	33.087	17.991	19.012	19.247
Case No. 3	31.592	15.776	16.532	16.699
Case No. 4	29.302	23.325	24.256	24.436
Case No. 5	29.047	34.308	35.055	34.756
Case No. 6	33.122	21.819	19.108	25.586
Case No. 7	28.718	22.249	18.069	28.168
Case No. 8	27.191	29.079	29.185	35.269
Case No. 9	28.711	19.096	19.298	19.989
Case No. 10	28.847	29.571	30.486	23.025
Case No. 11	26.190	30.674	31.953	Undetectable
Case No. 12	24.731	30.403	24.160	32.997
Case No. 13	26.902	13.322	12.022	16.587
Case No. 14	26.429	33.384	32.192	35.215
Case No. 15	26.954	28.723	30.657	26.265

**Table 3:** Immunohistochemistry results in the study group using the anti-SARS-CoV-2 nucleocapsid antibody.

	LUNG									
	Pneumocytes				Macrophages		Fibroblasts		Hyaline Membranes	
	>1 Cell/Field		<1 Cell/Field		Weak	Intense	Weak	Intense	Weak	Intense
CASE NO.	Weak	Intense	Weak	Intense						
1	0	1	0	0	0	1	0	0	0	0
2	0	1	0	0	0	0	0	0	0	1
3	0	1	0	0	0	1	0	0	0	0
4	0	0	0	1	1	0	1	0	0	0
5	0	0	0	1	0	1	0	1	0	0
6	0	1	0	0	0	0	0	0	0	0
7	0	1	0	0	0	0	0	0	0	1
8	0	0	1	0	1	0	1	0	0	0
9	0	0	0	0	0	0	0	0	0	0
10	0	0	0	0	0	0	0	0	0	0
11	0	0	1	0	0	1	0	0	0	0
12	0	1	0	0	0	1	0	1	0	1
13	0	0	0	0	0	0	0	0	0	0
14	0	1	0	0	1	0	1	0	1	0
15	0	0	0	1	0	1	0	1	0	1
TOTAL	0	7	2	3	3	6	3	3	1	4
	7		5							
	12				9		6		5	
	12									

**Table 4:** Immunohistochemistry results in the study group using various antibodies.

	CD3	CD5	CD20	CD68	CK7	TTF1	CK AE1/AE3	CK MNF 116
T Lymphocytes	+++	+++	-	-	-	-	-	
B Lymphocytes	-	-	+	-	-	-	-	-
Macrophages	-	-	-	+++	-	-	-	-
Type II pneumocytes	-	-	-	-	+++	+++	-	++
Cells with viral cytopathic effects	-	-	-	-	++	++	-	++
Multinucleated Giant cell-like aggregates	-	-	-	-	+	+	-	+
Hyaline membranes	-	-	-	-	-	-	+++	+++

+ Cells/tissues positive for this immunostaining were found scattered throughout the samples. ++ Cells/tissues positive for this immunostaining were found in moderate quantities throughout the samples. +++ Cells/tissues positive for this immunostaining were found in abundance throughout the samples. - Cells/tissues are negative for this immunostaining.

## Data Availability

Data are contained within the article.
